# COVID-19 as a Syndemic

**DOI:** 10.3389/fpubh.2021.763830

**Published:** 2021-09-09

**Authors:** Emilie Courtin, Paolo Vineis

**Affiliations:** ^1^London School of Hygiene and Tropical Medicine, London, United Kingdom; ^2^MRC Centre for Environment and Health, Imperial College London, London, United Kingdom

**Keywords:** COVID-19, SARS-CoV-2, blood glucose, syndemic, socially transmitted disease

There are several reasons why the paper by Logette et al. (1) on the complex metabolic pathways underlying the clinical course of COVID-19 is innovative and important. They developed machine learning models and mined as many as 240,000 open access scientific papers. Their results point to glucose metabolism as a key pathway in the clinical progression of COVID-19. Their interpretation, that needs to be further verified by empirical research, is that elevations of glucose provide ideal conditions for the virus to weaken the immune defenses in the lungs and bind to the ACE2 receptor, inducing a pulmonary inflammatory response. Strengths and limitations of their approach are described by the authors themselves and the two accompanying commentaries by Holly and Hartung.

One of the reasons for the importance of the paper is that it sheds light on the concept of syndemic, and its relevance in the context of the COVID-19 pandemic. This concept was first coined by anthropologist Merrill Singer to describe the AIDS epidemic and its drivers, focusing in particular on the co-occurrence of substance abuse, violence, and AIDS (SAVA) (1). Emily Mendenhall has since extended the concept to chronic diseases in her description of the VIDDA syndemic: violence, immigration, depression, type 2 diabetes, and abuse among Mexican immigrant women in the US (2). The goal of the syndemic model is to examine the health consequences of identifiable disease interactions and the social/environmental/economic factors that promote such interactions and ultimately worsen disease outcomes. The questions that drove the development of this model are key to further our understanding of COVID-19 and its drivers: (1) why certain diseases cluster (defined as multiple diseases affecting the same individuals or group); (2) what are the pathways through which these diseases interact biologically and end up multiplying their overall disease burden; (3) how do social environments (and in particular social inequalities) contribute to disease clustering and interactions?

Researchers and commentators have called for COVID-19 to be considered a syndemic (3, 4). The notion seems to fit well with the emerging evidence on inequalities in vulnerability, susceptibility, exposure and transmission of the infection. Individual-level clinical factors such as age, sex, obesity, and chronic conditions have been associated with higher rates of hospitalisations and deaths (5–9). Socio-economic factors are also drivers of variations in COVID-19 morbidity and mortality: COVID-19 mortality in the most deprived areas of England and Wales was almost twice the rate of the least deprived (10). Low-income, ethnic-minority groups are more likely to be exposed to the virus, to succumb to it, and are less able in multi-generational, overcrowded housing to protect their family members from the disease (11). In addition, low-income populations are more impacted by diseases related to premature aging (e.g., diabetes, hypertension, high cholesterol) (12, 13).

Importantly, multiple mechanisms of susceptibility to the effects of infection with Sars-CoV-2 have started to be uncovered. For example, it has been shown that kynurenine-related metabolism, and particularly the levels of kynurenic acid (KA), are predictors of COVID-19 progression and prognosis (14). Kynurenic acid and a high KA – to – kynurenine (K) ratio (KA:K) were positively associated with age and with inflammatory cytokines and chemokines, and negatively associated with T cell responses. A key player in this metabolic pathway is the Ah receptor, of which KA is the natural ligand. Activation of the kynurenine pathway and of AhR is involved in a multiplicity of environmentally-induced non-communicable diseases (NCD) and could explain why COVID-19 can act as a syndemic.

Logette's paper is focused on the clinical course of COVID-19, highlighting the role of glucose metabolism in triggering the inflammatory response to the virus. From this point of view the paper does not address etiology and the concept of syndemic directly. However, it is clear that conditions such as the metabolic syndrome, obesity, and diabetes are associated with the likelihood of infection (particularly the symptomatic infection) and also with the overarching role played by socio-economic circumstances, a strong predictor of infection and its clinical course. The focus on glucose is not incompatible with other mechanisms being at work in increasing susceptibility to Sars-CoV-2 and explaining why COVID-19 may be interpreted as a syndemic. Richard Horton has drawn our attention to the fact that fighting the pandemic implies in fact fighting non-communicable diseases, or, as we argue below, socially-transmitted diseases (4).

## Socially-Transmitted Diseases and Syndemics

If we look at the landscape of non-communicable diseases today, they are characterized by the features listed by Allen and Feigl (15): chronicity, global burden, preventable nature, shared proximal risk factors (cholesterol, blood pressure, glucose, obesity), common behavioral risk factors (smoking, alcohol, diet, inactivity, air pollution), common distal risk factors (economic, social, environmental), common issues of inequality and injustice. On this basis, Allen and Feigl suggest to call them “socially-transmitted diseases”. Let us examine the features of syndemics in the light of Allen and Feigl's proposal.

*First*, co-occurrence of diseases are at the core of the syndemic concept. The prevalence of multi-morbidity, defined as the occurrence of two or more chronic diseases in an individual may range from 20 to 40% in middle age, and up to 80% in older ages. In addition to co-occurrence in general, there is ample evidence of multiple outcomes related to single risk factors, e.g., air pollution or smoking inducing cardiovascular diseases, respiratory diseases, and cancer.

*Second*, what are the mechanistic pathways leading to such co-occurrence? We are still at the stage of a patchwork with multiple candidates that are not yet clearly connected to each other, and belong to different biological layers. Good candidates are inflammatory pathways, such as “inflammaging” (including the kynurenine pathways described above), and, at another level, epigenetic pathways. A related concept is biologic clocks or “age acceleration”, measurable with epigenetic tools. Whether an individual will follow a trajectory of accelerated or decelerated aging will depend on their genetic background interacting with life-long environmental and lifestyle factors.

*Third*, the social component of syndemics. Aging is characterized by a gradual and constant increase in health inequalities among individuals. For example, considerable differences in healthy aging have been described among socioeconomic groups, an association which is only partially explained by unhealthy lifestyle habits of more disadvantaged individuals. In recent years, various measures of biological aging have been proposed, such as telomere length, allostatic load, and biological clocks developed using high-throughput “omic” data (epigenomic, transcriptomic, metabolomic, etc.). All of these were associated with measures of social inequalities (16). The central role of social inequalities in aging and disease risk suggests that the concept of syndemic can be broadly applicable to NCD.

In summary, whether or not COVID-19 is a syndemic remains to be demonstrated. Much attention has been paid (rightly so) to social inequalities and co-occurrence with other diseases, less to underlying mechanisms. The three components of the concept of syndemic have a clear bearing on practical preventive action, specifically preparedness. In this context, Logette's paper contributes substantially to build bridges between NCD and infectious diseases and thus facilitates preparedness to future pandemics.

## Author Contributions

All authors listed have made a substantial, direct and intellectual contribution to the work, and approved it for publication.

## Funding

EC acknowledges funding from the Medical Research Council (MR/T032499/1).

## Conflict of Interest

The authors declare that the research was conducted in the absence of any commercial or financial relationships that could be construed as a potential conflict of interest.

## Publisher's Note

All claims expressed in this article are solely those of the authors and do not necessarily represent those of their affiliated organizations, or those of the publisher, the editors and the reviewers. Any product that may be evaluated in this article, or claim that may be made by its manufacturer, is not guaranteed or endorsed by the publisher.
